# Nuclear phylogeography of the temperate tree species *Chiranthodendron pentadactylon* (Malvaceae): Quaternary relicts in Mesoamerican cloud forests

**DOI:** 10.1186/s12862-020-01605-8

**Published:** 2020-04-19

**Authors:** Diana Gabriela Hernández-Langford, María Elena Siqueiros-Delgado, Eduardo Ruíz-Sánchez

**Affiliations:** 1 Departamento de Biología, Herbario UAA, Centro de Ciencias Básicas, Edificio 132, Universidad Autónoma de Aguascalientes, Av, Universidad No. 940, Ciudad Universitaria, 20131 Aguascalientes, Aguascalientes México; 2 Departamento de Botánica y Zoología, Centro Universitario de Ciencias Biológicas y Agropecuarias, Universidad de Guadalajara, Camino Ing. Ramón Padilla Sánchez 2100, Nextipac, 45200 Nextipac, Zapopan, Jalisco México

**Keywords:** Cloud forests, Guatemala, Isthmus of Tehuantepec, Mexico, Pine-oak forests, Quaternary relicts

## Abstract

**Background:**

The *Mexican hand tree* or *Canac* (*Chiranthodendron pentadactylon*) is a temperate tree species of cloud and pine-oak forests of southern Mexico and Guatemala. Its characteristic hand-shaped flower is used in folk medicine and has constituted the iconic symbol of the *Sociedad Botánica de México* since 1940. Here, the evolutionary history of this species was estimated through phylogeographic analyses of nuclear DNA sequences obtained through restriction site associated DNA sequencing and ecological niche modeling. Total genomic DNA was extracted from leaf samples obtained from a representative number (5 to 10 per sampling site) of individuals distributed along the species geographic range. In Mexico, population is comprised by spatially isolated individuals which may follow the trends of cloud forest fragmentation. By contrast, in Guatemala *Chiranthodendron* may constitute a canopy dominant species near the Acatenango volcano. The distributional range of this species encompasses geographic provinces separated by the Isthmus of Tehuantepec.

The objectives of the study were to: (i) estimate its genetic structure to define whether the observed range disjunction exerted by the Isthmus of Tehuantepec translates into separate populations, (ii) link population divergence timing and demographic trends to historical climate change, and (iii) test hypotheses related to Pleistocene refugia.

**Results:**

Patterns of genetic diversity indicated high levels of genetic differentiation between populations separated by the Isthmus. The western and eastern population diverged approximately 0.873 Million years ago (Ma). Demographic analyses supported a simultaneous split from an ancestral population and rapid expansion from a small stock approximately 0.2 Ma corresponding to a glacial period. The populations have remained stable since the LIG (130 Kilo years ago (Ka)). Species distribution modelling (SDM) predicted a decrease in potential distribution in the Last Interglacial (LIG) and an increase during the Last Glacial Maximum (LGM) (22 Ka), Mid-Holocene (6 Ka) and present times.

**Conclusions:**

Divergence time estimations support the hypothesis that populations represent Quaternary relict elements of a species with broader and northernmost distribution. Pleistocene climatic shifts exerted major influence on the distribution of populations allowing dispersion during episodes of suitable climatic conditions and structuring during the first interglacial with a time period length of 100 Kilo years (Kyr) and the vicariant influence of the Isthmus. Limited demographic expansion and population connectivity during the LGM supports the moist forest hypothesis model.

## Background

*Chiranthodendron pentadactylon* Sessé ex. Larreat is an evergreen temperate tree species of tropical montane cloud forests and pine-oak forests of southern Mexico [1] (Guerrero, Oaxaca and Chiapas) and Guatemala [2] (El Progreso, Zacapa, Sacatepéquez, Chimaltenango, Sololá, Totonicapán, Quetzaltenango, Quiché, Huehuetenango, and San Marcos). It is distinguished by dark reddish flowers in which the androecium portion that harbors the stamens resembles the fingers of a hand hence the vernacular names of *Mexican hand tree* or *tree of the little hands* in Mexico and *Canac* (hand-flower) in Guatemala. The flowers are currently used in folk medicine to treat heart conditions, high pressure [3] and gastrointestinal disorders such as diarrhea and dysentery [4]. Furthermore, the flower has constituted the iconic symbol of the *Sociedad Botánica de México* since the early 40s. In Mexico, the population spatial structure is constituted by spatially isolated individuals which may follow the trends of cloud forest fragmentation. On the other hand, Véliz [2] observed that *C. pentadactylon* represents a canopy dominant species of the cloud forest community found near the Acatenango volcano in Guatemala.

In the IUCN Red List of Threatened Species [1], *C. pentadactylon* is listed as vulnerable in Mexico and as near threatened in Guatemala [5]. The *Norma Oficial Mexicana* (NOM-059-SEMARNAT-2010) [6] however has listed *C. pentadactylon* as threatened. This implies that populations could become endangered in the short or medium term if the factors that negatively affect their viability continue to operate including deterioration or modification of their habitat or direct reduction of their population sizes [6].

The monospecific genus *Chiranthodendron* Larreat. along with *Fremontodendron* Coville, form the Fremontodendreae tribe within the Bombacoideae subfamily of the Malvaceae family. *Fremontodendron* bears three species: *F. californicum* (Torrey) Coville, *F. mexicanum* Davidson and *F. decumbens* R. M. Lloyd. They are shrubs and small trees that constitute distinctive elements of the chaparral vegetation in the Sierra Nevada foothills and coastal ranges of California extending to scattered locations in central and western Arizona and northern Baja California in Mexico [7]. *Chiranthodendron* and *Fremontodendron* share leaf-opposed hermaphroditic flowers greater than 2 cm in diameter and the production of large amounts of nectar [8].

The high biotic diversity of Mesoamerican forests [9, 10] is the result of the interchange between Nearctic and Neotropical floristic components [10–13]. This interchange has taken place during different time periods of climate change and depends on both historical and ecological factors. For instance, pollen records from the Elsinore flora in San Diego, suggest a cooling and drying trend beginning in the middle Eocene (52–40 Ma) and Late Eocene (40–38 Ma) [14] to early Miocene (23.3–16.3 Ma) [15] which resulted in the establishment of a winter-dry mixed deciduous hardwood-coniferous forest in the uplands, and the replacement of the Paleocene (65–56.5 Ma) to earliest Lutetian (48.6 Ma) tropical and paratropical lowland vegetation by more seasonally dry communities.

The drying trend is evident from pollen records suggesting the last appearance of *Chiranthodendron*-*Fremontodendron* potential predecessors from the early Lutetian. Hence, species with north temperate affinity constitute forms that survived the progressive drying and cooling trends of the middle Eocene and early Miocene. Subsequent colonization events have ensued during the Pliocene epoch (5.33–2.59 Ma) whence the cooling trend subsided gradually culminating with higher temperatures and wetter climatic conditions in the mid-Pliocene (3.6 Ma). Thereupon, during the late Pliocene (3 Ma) temperatures decreased leading to the onset of the Pleistocene (2.59–0.01 Ma) glacial cycles with the formation of the North American Laurentide sheet approximately 2.6 Ma [15]. These have been regarded as more recent drivers of species diversification and plant distribution dynamics [16–22]. Species responses to the Pleistocene ice ages include extinction over a large part of their range, dispersion to new locations, survival in refugia and postglacial colonization [23]. The extent of change in species ranges depended on latitude and topography. High latitudes were covered with ice or permafrost while temperate and tropical regions were compressed towards the equator [22].

The genetic and demographic consequences suggested for Mesoamerican cloud forest species have been interpreted under refugia hypotheses, which describe differing precipitation regimes. These include the moist forest hypothesis and the dry refugia model. The moist forest hypothesis states that during glacial periods there was no significant change in precipitation allowing the development of population connectivity, downslope migration and range expansion [23]. The degree of genetic structure homogenization and population growth is positively correlated with the species dispersal abilities [24]. During dry and warm interglacial periods there was upslope migration, range contraction, isolation and genetic differentiation [23]. Conversely, the dry refugia model suggests a significant reduction in precipitation during glacial periods in which there was downslope migration but no population connectivity or gene flow among populations. Populations were compressed into refugia by the opposing effects of aridity and cooling subsequently becoming isolated and genetically differentiated. During humid and warm interglacial periods, these populations expanded and recolonized their former range. The degree of population differentiation depended on the extent of former refugia and on their past levels of gene flow [23].

Presently, Mesoamerican cloud forests take place in narrow altitudinal zones [25] and occur in restricted and limited areas [26]. They represent evergreen forests [27] characterized by a constant, frequent or seasonal cloud cover [25]. The geographic range of *Chiranthodendron* covers several geographic provinces separated mainly by the Isthmus of Tehuantepec and may represent a potential physical and ecological barrier [28] to dispersal and gene flow. Therefore, lineages west and east of the Isthmus might constitute separate populations. However, their current distribution may be the result of the influence of past climate changes as opposed to mainly the potential vicariant effect of the Isthmus. Furthermore, the long lifespan of trees hampers their rapid adaptation to environmental changes [29] which make them particularly vulnerable to climate changes influencing their population dynamics, genetic diversity patterns and demographic history.

To gain insight into the potential historical factors influencing *C. pentadactylon* present distribution, the evolutionary history is examined through phylogeographic, population genetic and ecological niche modelling approaches. The main objectives of the study were to use *C. pentadactylon* to: (i) estimate the genetic structure to define whether the observed range disjunction exerted by the Isthmus of Tehuantepec translates into separate populations, (ii) link population divergence timing and demographic trends to historical climate change, and (iii) test hypotheses related to Pleistocene refugia.

Genetic analyses were conducted using nuclear loci obtained through restriction-site associated DNA sequencing (RADSeq). Nuclear genomic DNA may be the source of numerous unlinked loci of enough resolution to detect spatial distributional patterns of gene genealogies [30]. Moreover, nuclear markers are biparentally inherited reflecting both seed and pollen movement [31] as opposed to the traditionally used chloroplast DNA markers [11, 30] which reflect only seed movement [31] and the spatial genetic diversity patterns are thus only partially analyzed.

## Results

### Genetic diversity and genetic differentiation

**Fig. 1:**
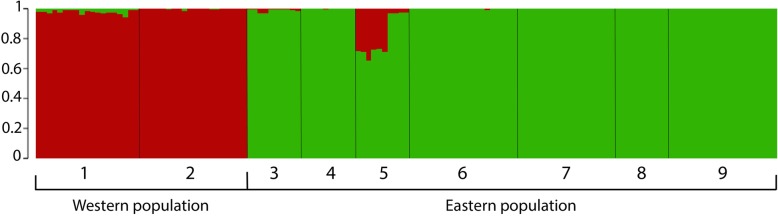
STRUCTURE bar plot showing the individual membership coefficients assigned to each K number of groups (K = 2) defined as populations. The individuals are classified according to their sampling site: 1) Carrizal de Bravo, Guerrero. (2) San Mateo Río Hondo, Oaxaca. (3) San Cristóbal de las Casas, Chiapas. (4) Motozintla, Chiapas. (5) Tacaná volcano, Chiapas. (6) Sierra de los Cuchumatanes, Huehuetenango. (7) Totonicapán. (8) Chimaltenango-Quetzaltenango. (9) Acatenango volcano, Chimaltenango

The overall nucleotide diversity (Pi) was low (0.01463). The STRUCTURE analysis yielded a K value of 2 for the number of groups (Fig. 1) with the highest likelihood (Additional file 5). The first group includes the individuals sampled west of the Isthmus of Tehuantepec (Guerrero and Oaxaca) and the second group includes the individuals sampled east of the Isthmus (Chiapas and Guatemala). This arrangement of groups was further introduced in Arlequin to evaluate the degree of genetic differentiation through a global analysis of molecular variance (AMOVA). The AMOVA (Table 1) showed that most of the variation is attributed to differences among groups (62.87%) with a fixation value (F_ST_) of 0.62879 followed by moderate levels of within population variation (37.12%). The estimated degree of genetic differentiation among groups separated west and east of the Isthmus supports their attribution as separate populations referred to as western and eastern population respectively.

**Table 1:** Results of global AMOVA based on SNPs nuclear sequences. The populations west and east of the Isthmus of Tehuantepec defined by STRUCTURE were established a priori as separate groups

Global AMOVA	Sum of squares	Variance components	Percentage of variation	Fixation indexes
Among populations	509.750	9.29102	62.87942	F_ST_ = 0.62879
Within populations	733.194	5.48491	37.12058	
Total	1242.944	14.77593		*P* < 0.000

### Phylogenetic network and divergence times

**Fig. 2:**
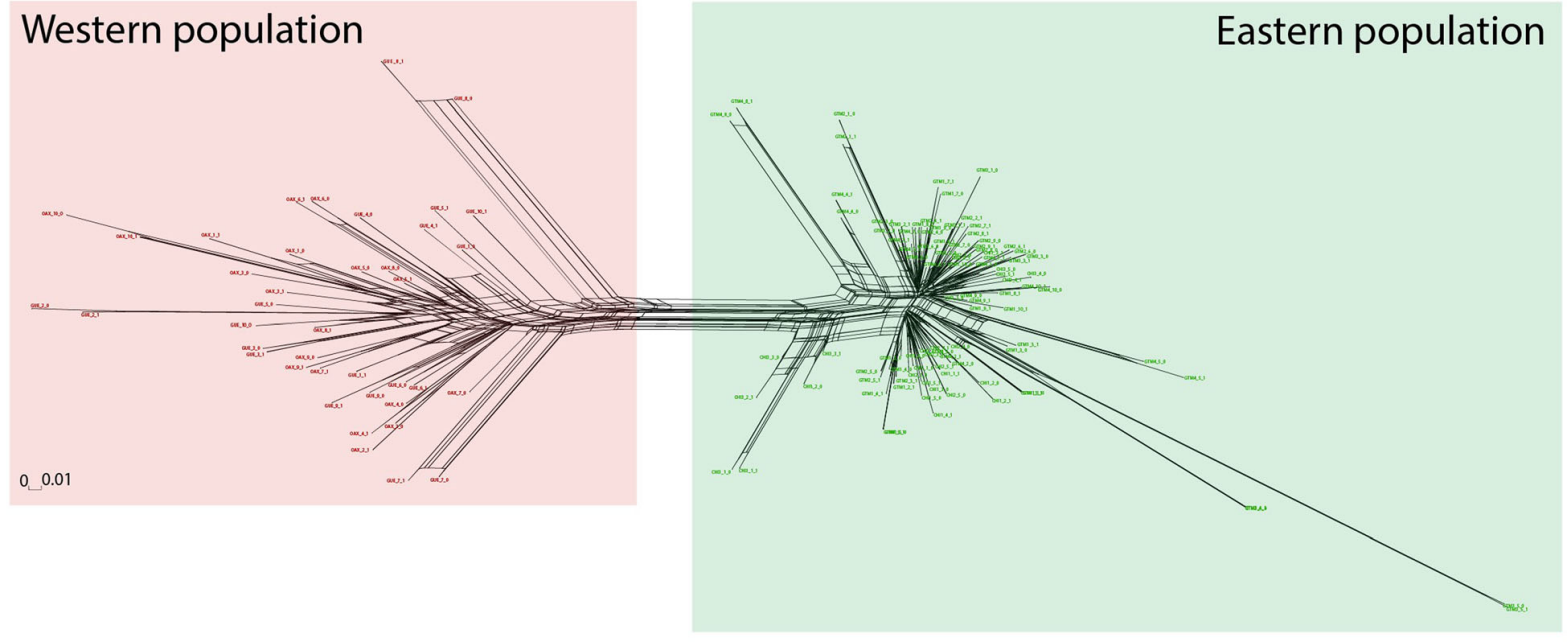
Phylogenetic network (Neighbor-Net) constructed by SplitsTree based on SNPs nuclear sequences. The recovered clades correspond to the western and eastern populations defined by STRUCTURE

**Fig. 3:**
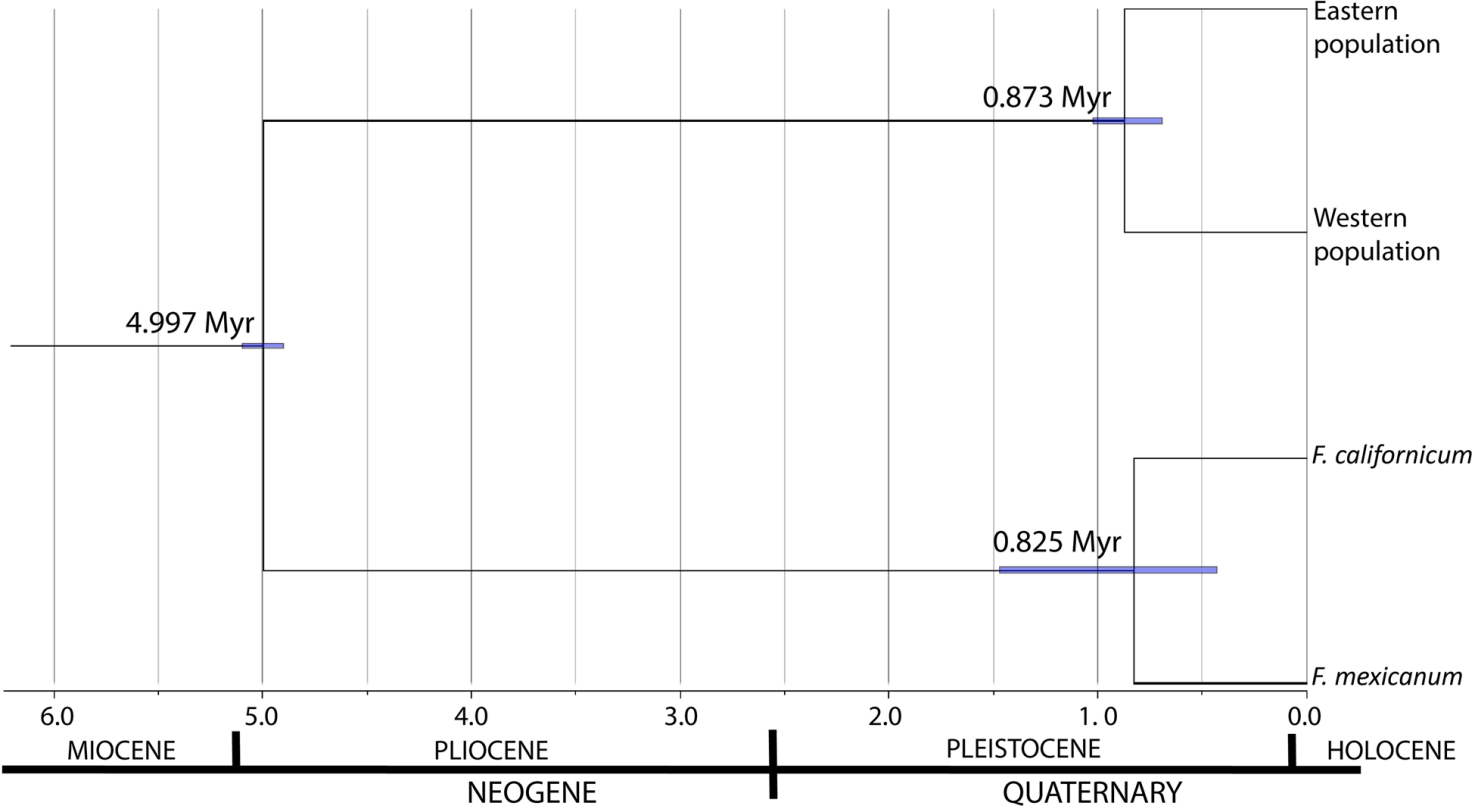
Chronogram of *C. pentadactylon* populations based on consensus tree using 36 polymorphic nuclear loci and a multispecies coalescent model with constant size. The 95% Highest Posterior Density (HPD) intervals are displayed as purple bars. The root of the tree was calibrated using a secondary calibration point of 5 Myr. The estimated divergence time between western and eastern population is on the order of 0.873 Myr

The phylogenetic network is shown in Fig. 2 and shows the same set of populations inferred by STRUCTURE. The StarBEAST2 tree (Fig. 3) showed a strong support (PP = 1) for the differentiation of the clades west and east of the Isthmus of Tehuantepec. The western and eastern population diverged approximately 0.873 Ma (HPD 95%: 0.693–1.022 Ma).

### Demographic history

**Fig. 4:**
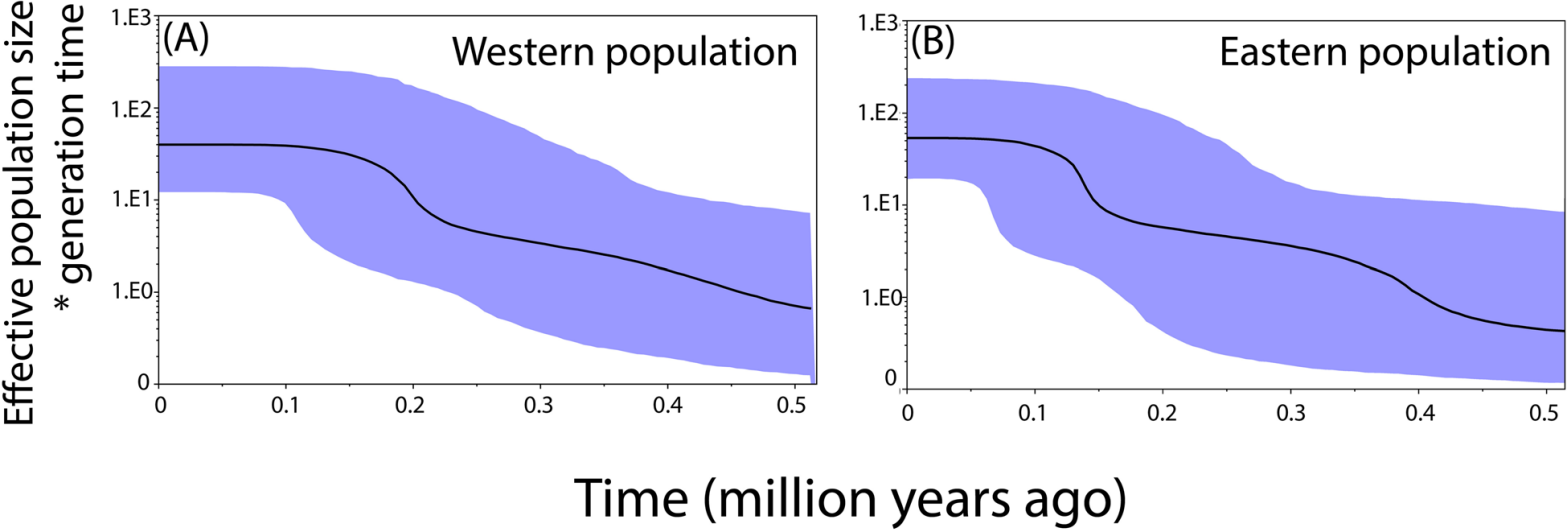
Bayesian skyline plots estimated from nuclear SNPs sequences showing the historical demographic trends for each population: A) Western population (Guerrero & Oaxaca), B) Eastern population (Chiapas & Guatemala). The y axis corresponds to the product between the effective population size and the generation time and the x axis to the time elapsed in million years. Median solid lines represent mean estimates and the shaded areas correspond to 95% confidence intervals

The BSPs estimated for the SNPs sequences indicated an ongoing increase in effective population size from 0.5 Ma onwards followed by sudden demographic expansions. The western population (Fig. 4a) underwent one sudden demographic expansion approximately 0.2 Ma while the eastern population (Fig. 4b) two sudden demographic expansions, the first approximately 0.4 Ma and the second approximately 0.15 Ma. After the latest expansions, effective population sizes have remained stable until recent times.

**Fig. 5:**
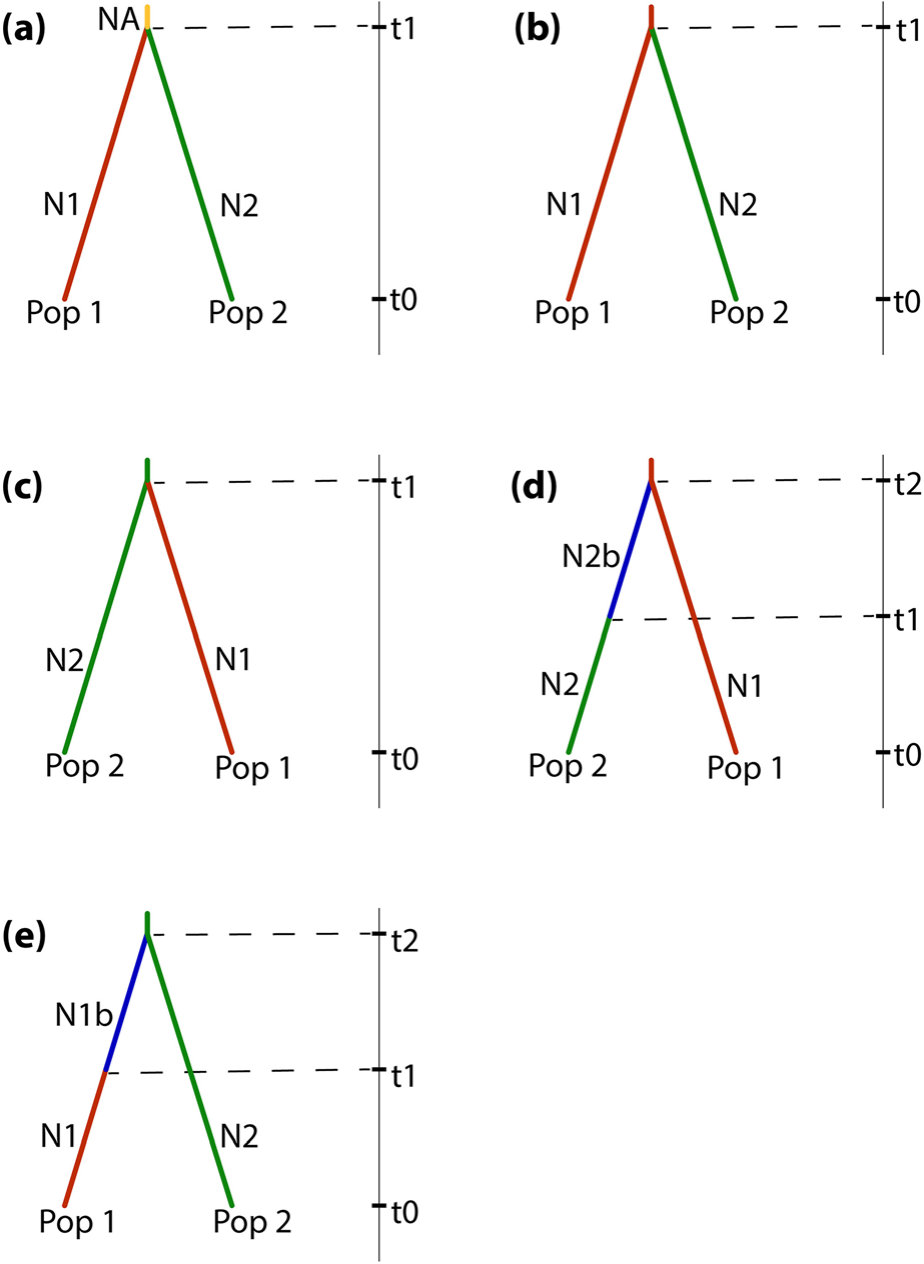
Competing demographic scenarios of *Chiranthodendron pentadactylon* simulated through DIYABC based on SNPs sequences. **a** Scenario 1: Simultaneous split from an ancestral population at t1. **b** Scenario 2: Eastern population (Pop 2) diverged from western population (Pop 1) at t1. **c** Scenario 3: Western population diverged from eastern population at t1. **d** Scenario 4: Eastern population derived from few western immigrants at t2 and diverged at t1. **e** Scenario 5: Western population derived from few eastern immigrants at t2 and diverged at t1

Considering the five scenarios (Fig. 5) tested for the SNPs sequences, DIYABC analysis indicated that a simultaneous split from an ancestral population (scenario 1) is the best-supported scenario with a higher posterior probability value than estimated for other scenarios (Table 2). Confidence in scenario choice evaluation based on 500 PODs yielded low values for the Type I and Type II errors.

**Table 2:** Posterior probability of each scenario and 95% confidence intervals (CI) based on the logistic regression approach for approximate Bayesian computation (ABC). Type I and type II errors were estimated for the scenario with the highest posterior probability (Scenario 1)

Scenario	Posterior probability	95% CI	Type I error	Type II error
1. Simultaneous split from an ancestral population.	0.8400	0.7906–0.8895	0.0260	0.2565
2. Eastern population diverged from western population.	0.0011	0.0000–0.2607		
3. Western population diverged from eastern population.	0.0039	0.0000–0.2632		
4. Eastern population derived from few western immigrants.	0.0035	0.0000–0.2629		
5. Western population derived from few eastern immigrants.	0.1515	0.1046–0.1984		

### Distribution modelling

**Fig. 6:**
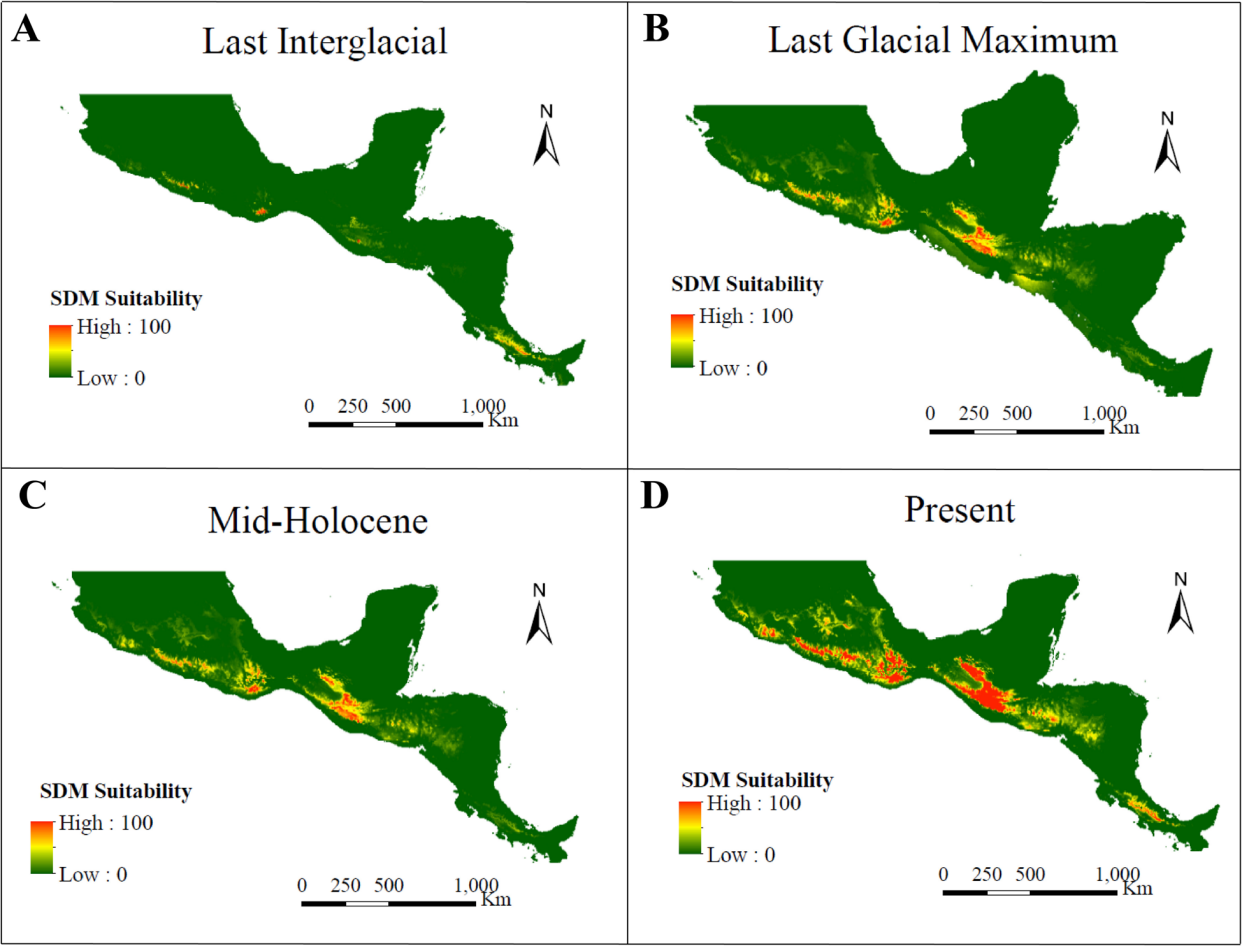
Current and historical distribution predicted through ecological niche modeling implemented by the maximum entropy method. The historical distributions ages are **a**) Last Interglacial, 130 Kyr, **b**) Last Glacial Maximum, 22 Kyr and **c**) Mid-Holocene, 6 Kyr. The probability of occurrence based on climate suitability (SDM Suitability) is shown from high (100) in red to low (0) in green. Maps by DGHL

The mean area under the receiver operating characteristic curve (ROC) for the replicate runs displays a good model performance (0.970). The species distribution modelling (SDM) for the LIG shows a major reduction in the species ecological niche as compared with the SDM for the LGM and Mid-HLC in which the species ecological niche is increased. The potential geographic distribution is maintained in the LGM through the Mid-HLC but is further increased in recent times (Fig. 6).

## Discussion

### The vicariant role of the isthmus of Tehuantepec in shaping patterns of genetic diversity

The distributional range of *C. pentadactylon* encompasses geographic provinces separated by the Isthmus of Tehuantepec. The Isthmus of Tehuantepec is a savannah-like valley area [32] of high seismicity that unites the North American continent with Nuclear Central America. At the North American continent junction decreases to 200 Km width [33]. Geological evidence from the late Miocene to late Pliocene suggests a reduction of the highlands and possible marine embayment as consequence of an extensive down dropping of the eastern block of the Tehuantepec fault [32, 33]. The Isthmus of Tehuantepec has been regarded as a biogeographical barrier to many taxa. Studies on Mesoamerican cloud forest species currently codistributed alongside and near the Isthmus of Tehuantepec in Mexico, observed a genetic and distributional range disjunction produced by the occurrence of the Isthmus. For instance, phylogeographic studies of the distylous shrub *Palicourea padifolia* [34] and of the mistletoe cactus *Rhipsalis baccifera* which followed the trends of cloud forest demographic dynamics during periods of climate change [35] revealed a genetic and population disjunction at the Isthmus. Moreover, a comparative phylogeographic study that included the cloud forest tree species *Podocarpus matudae* and *Liquidambar styraciflua*, the shrub *P. padifolia*, the herb *Moussonia deppeana* and the epiphytic *Rhipsalis baccifera* as well as the rodent species *Peromyscus aztecus, Reithrodontomys sumichrasti,* and *Habromys lophurus*, the hummingbird species *Amazilia cyanocephala, Campylopterus curvipennis* and *Lampornis amethystinus,* the woodcreeper *Lepidocolaptes affinis* and the passerine species *Chlorospingus opthtalmicus, Buarremon brunneinucha* and *Basileuterus belli* [32] likewise supported a genetic and population disjunction at the Isthmus. Conversely, in the mistletoe *Psittachanthus schiedeanus* [36] the vicariant role of the Isthmus was not evident due to effective gene flow mechanisms resulting in shallow levels of genetic structure.

To determine whether the distribution disjunction exerted by the Isthmus of Tehuantepec represents a biogeographical barrier to dispersal and gene flow, population estimates were conducted based on nuclear data derived from RAD sequencing. The degree of population substructure was estimated under the Bayesian approach implemented by the software STRUCTURE. The Bayesian algorithm detected a genetic differentiation among *C. pentadactylon* lineages distributed west and east of the Isthmus of Tehuantepec and were thenceforth considered as separate populations. The degree of genetic differentiation among populations was further estimated by the global AMOVA implemented by Arlequin which indicated high levels of genetic differentiation among populations and moderate levels of within population variation. The high vagility of the pollination vectors such as birds and bats might induce low levels of among population variation. Nevertheless, the probability of long-distance dispersal events along the Isthmus is reduced given the hermaphroditic floral morphology and therein breeding system. On the other hand, the dichogamous nature of the flowers promotes a mixed-mating mechanism which derives in moderate levels of within population genetic variation. Further, low levels of within population subdivision are expected given the highly vagile pollination vectors such as birds and bats since they are likely to visit numerous plants [37]. Seed dispersal mediated by birds might also result in low levels of within population subdivision.

Finally, the relationships among individuals estimated by the Neighbor-Net algorithm consisted on the two populations detected by previous analyses. Moreover, the StarBEAST2 tree showed a strong support for the differentiation of the populations.

### Population divergence timing and demographic history relations to historical climate change

Changes in the distribution of cloud forests throughout time have been determined by factors such as geological events and climate change. In addition to the occurrence of the Isthmus of Tehuantepec, the geographic range of *C. pentadactylon* might have been influenced by the climatic oscillations of the Quaternary ice ages. To relate genetic divergence to pre-Pleistocene and Pleistocene events, calibrated time trees were calculated under the multispecies coalescent model implemented in StarBEAST2. *Chiranthodendron* and its sister genus *Fremontodendron* bring together the Fremontodendreae tribe within the Bombacoideae subfamily. These might have derived from predecessors with Laurasian origin [38] which migrated to North America and subsequently to Mesoamerica [39]. According to Richardson et al. [40], the MRCA of the Fremontodendreae tribe dates to approximately 5 Ma in the early Pliocene. Using this date as calibration point and 36 polymorphic nuclear loci, populations divergence was estimated to have a median age of 0.873 Myr. This date approximates to the latest stage of a slow cooling progress developed since the last 50 Myr. At the start of the Pleistocene epoch, alternations between cold glacial periods and warmer intervals took place and between 1.2 and 0.6 Ma weaker cycles with a period of 40 Kyr gave way to stronger cycles of approximately 100 Kyr mostly beginning 800 Ka [41]. The extended warming of this time period may have reduced the potential distribution of *Chiranthodendron* and contracted its range to the highlands where suitable climatic conditions remained. The DIYABC analysis based on SNPs sequences supported a simultaneous split from an ancestral population scenario. This scenario suggests a once widespread ancestral species that contracted its range during a period of unsuitable climatic conditions as those inferred for the last 800 Kyr leading to variation in population continuity and isolation. Range contraction might have included a northernmost distribution. This is supported by the upheld distribution of its sister genus *Fremontodendron* in southwestern United States and northwestern Mexico. This genus is comprised by shrubs and small trees of chaparral communities [7] and dry temperate sclerophyllous floras [42]. Some predecessors might have survived the climatic shifts of the Pleistocene epoch and adapted successfully to these climatic conditions. While some dispersed to northwestern Mexico possibly in the early Pleistocene during glacial intervals from which low-pressure systems allowed a deeper penetration of cyclones from the Pacific Ocean and the Gulf of Mexico [15]. Later, some predecessors may have reached southern Mexico and Guatemala. The distribution might have become restricted to a southernmost distribution after more severe cold periods beginning approximately 1.1 Ma [15] and the subsequent warming and drying trend of the late Holocene towards modern climatic conditions in northern Mexico [43].

Changes in population size over time were estimated through BSPs based on SNPs sequences. These analyses detected ongoing increases in effective population size from 0.5 Ma onwards followed by sudden demographic expansions. The latest demographic expansions might coincide with a glacial period dated approximately 0.2 Ma [41]. These expansions support the moist forest hypothesis in which downslope migration, range expansion and population connectivity are predicted [23]. High dispersal abilities might have allowed increases in population sizes [36] as opposed to the traditionally assumed little or no demographic expansion under this model. On the other hand, the low level of nucleotide diversity observed indicates demographic expansions from small size populations. Conversely, the absence of population size reductions or bottlenecks does not support their survival in refugia as expected under the dry refugia model in which populations were compressed into refugia by the opposing effects of aridity and cooling [23].

Phylogeographic studies on additional cloud forest species allowed the recognition of signatures of rapid demographic expansion. For instance, haplotype diversity and topology estimations coupled with neutrality tests suggested a rapid increase in population size from a small stock in the distylous shrub *P. padifolia* [34]. However, the high variation within populations as expected from species with high outbreeding rates, was not attributed to their survival in the small refugia proposed by Toledo [44] during the coldest periods of the Pleistocene epoch. Further studies on the geographic structure of genetic variation of highland populations of *Podocarpus spp.,* distributed in Mesoamerica along with the reconstruction of potential distributions during the LIG and LGM [24] allowed the recognition of demographic signatures related to the long-term in situ permanence in multiple refugia during the LGM which comprise the absence of demographic expansion and limited gene flow. The moist forest hypothesis model has also been inferred for *L. styraciflua* [9], *R. baccifera* [35], *P. schiedeanus* [36] and *Podocarpus* spp. [24] of which predicted distribution suggests a continuous spatial trend during the LGM with no significant demographic expansion.

In *C. pentadactylon*, reduction in potential distribution range predicted for the LIG may have prevented further demographic expansions but nevertheless reductions in effective population sizes were not observed. The latter suggests that populations might have survived in interglacial refugia and were able to maintain their effective population sizes. The absence of demographic expansions predicted for the LGM coincides with the demographic trends expected under the moist forest hypothesis.

The populations have remained constant from the LGM to present times even at the onset of an increase in potential distribution, suggesting the influence of additional variables such as ecological and anthropic factors.

## Conclusions

The phylogeographic approach conducted allowed the recognition and attribution of the Isthmus of Tehuantepec as a current driver of population differentiation and of the climatic oscillations of the Pleistocene epoch as a determining factor for their current distribution. Demographic analyses and divergence timing estimation suggest that populations derived from an ancestral population possibly of more widespread and northernmost distribution that underwent range contraction around the onset of the first interglacial with a time period length of 100 Kyr (800 Ka). Estimation of changes in population size over time suggests a demographic expansion during the glacial period prior to the LIG. The genetic diversity measures suggest an expansion from a small size population. The SDMs predicted a reduction in potential distribution for the LIG. However, population size reductions were not detected. Populations have not undergone demographic expansions since the unsuitable climatic conditions of the LIG and appear to remain stable. Nevertheless, the low genetic nucleotide diversity, low effective population sizes and restricted distribution to specific biomes limited in spatial range, make *C. pentadactylon* a species with limited adaptability to recent climate change and global warming.

Furthermore, the reduction of forest areas due to anthropic influence as established by the IUCN and the *Norma Oficial Mexicana* (NOM-059-SEMARNAT-2010), could render it endangered and consequently at risk of extinction. The disjunct distribution between *Chiranthodendron* and its sister genus *Fremontodendron* as well as the recent divergence time estimated for the extant populations, suggest that it represents a relict species and thus bears an additional value for conservation.

Additional ecological and evolutionary studies are required to determine the impact that *C. pentadactylon* provides to forest ecosystems, and to determine the characters subject to selection. This information may provide useful insights into the reproductive requirements and success of the species to efficiently conduct conservation efforts.

## Methods

### Study system

**Fig. 7:**
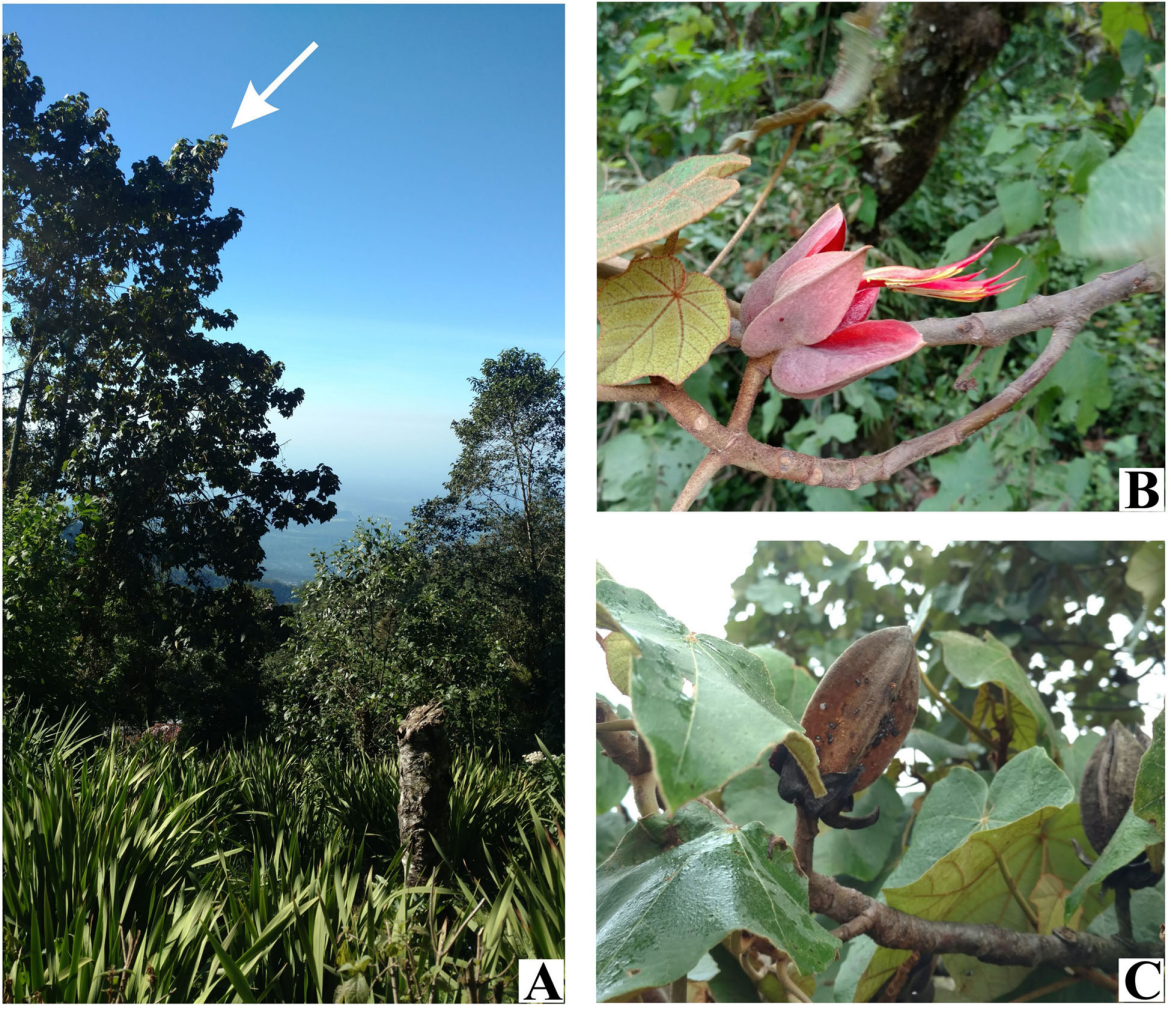
Mexican hand tree (*Chiranthodendron pentadactylon*)*,* near the Tacaná volcano, Chiapas (**a**). Lateral side view of flower (**b**). Dehiscent capsule fruit (**c**). Photographs by ERS

*Chiranthodendron pentadactylon* is a tree of up to 30 m tall (Fig. 7a) with a minimum generative growth time of more than 3 years [45]. It is resistant to low temperatures (down to 5 °C) and dry conditions [3]. The dichogamous red flowers (Fig. 7b) can be cross-pollinated or geitonogamously pollinated [46]. Pollination is mediated by birds [46] and bats [47]. The flowering period spans from November to April and the fruiting period from April to May [3]. Fruits are dehiscent capsules (Fig. 7c) and the strophiolated seeds [8] dispersal is potentially mediated by birds [46].

### Sampling and DNA sequencing

**Fig. 8:**
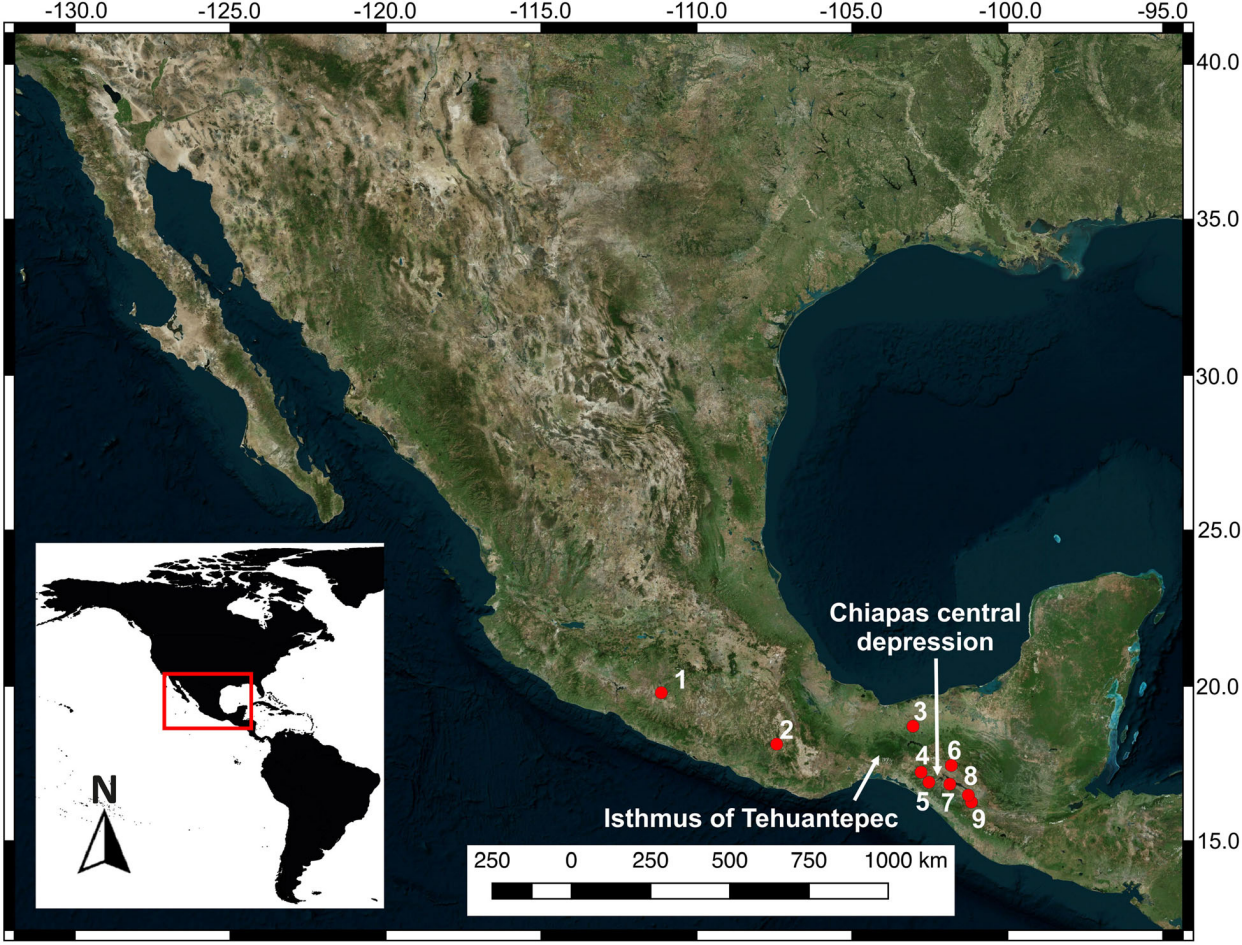
Geographical distribution of sampled localities for *C. pentadactylon* from Mexico and Guatemala. (1) Carrizal de Bravo, Guerrero. (2) San Mateo Río Hondo, Oaxaca. (3) San Cristóbal de las Casas, Chiapas. (4) Motozintla, Chiapas. (5) Tacaná volcano, Chiapas. (6) Sierra de los Cuchumatanes, Huehuetenango. (7) Totonicapán. (8) Chimaltenango-Quetzaltenango. (9) Acatenango volcano, Chimaltenango. Map by ERS

Leaf samples from 5 to 10 individuals per sampling site found along the range of *C. pentadactylon* in Mexico and Guatemala (Fig. 8) were collected and stored and dried with silica gel at room temperature until further processing. Two additional leaf samples from *Fremontodendron californicum* and *F. mexicanum* were collected from the Rancho Santa Botanic Garden in Claremont, California to be incorporated as outgroup species. No special permission was required to collect the samples. Taxon identification and geographic corroboration were performed during fieldwork after reviewing voucher specimens deposited in the herbarium of the Universidad Autónoma de Aguascalientes (Herbario UAA), the Instituto de Ecología AC at Pátzcuaro, the Facultad de Agronomía de la Universidad de San Carlos de Guatemala (AGUAT), the Escuela de Biología, USAC (Herbario BIGU), the Missouri Botanical Garden (MO) and the Chicago Natural History Museum. Each later defined population has an accompanying voucher deposited at the Herbarium UAA (Additional file 1).

Total genomic DNA was extracted following the cetyltrimethylammonium bromide (CTAB) protocol [48] with an additional incubation step with the enzyme pectinase to remove the excess of carbohydrates. Thereupon, a RAD sequencing library was prepared at Rancho Santa Ana Botanic Garden (Claremont, California) and at the University of California Riverside as described by Etter et al. [49] and modified by Medina [50] using *SbfI-HF* restriction enzyme (New England Biolabs). Samples were pooled into a multiplexed library and sequenced on an Ilumina NextSeq500 platform to generate 150-bp single-end readings.

The raw sequence data were analyzed and assembled de novo in the software pipeline ipyrad v0.7.28 [51], which identifies two sequences as orthologous as determined by a specified degree of similarity (0.90). Filtering parameters were set to trim all reads to a length of 140 bp. Reads were clustered allowing a maximum number of ambiguous base calls (Q < 20) of 0 and a maximum depth of coverage of 10 k. To date, karyotype information and ploidy level have not been determined, therefore loci containing more than two alleles were discarded to exclude potential paralogs.

When clustered across samples a minimum number of samples per locus were set to 66 to maximize the representativeness of the total samples per locus. The samples had an average of 565 K reads that passed quality filtering. These clustered into an average of 8837 clusters per sample with a mean depth of 4526 producing a mean of 4075 consensus sequences per sample and a maximum total of 70 loci were recovered (Additional file 2).

Loci retrieved from the RADSeq genome-partitioning approach are mainly from the nuclear genome [52]. However, 5 loci were highly similar to mitochondrial loci from closely related species as identified through the basic local alignment search tool (BLAST) option from the National Center for Biotechnology Information (NCBI) database and were discarded from further analysis, resulting in a total of 65 nuclear loci. Mitochondrial DNA substitution rate is at least 5 times slower than nuclear genes [53] and thence may not provide the enough resolution to detect infraspecific historical processes. Additional data files recovered from the alignment include the variant call format (vcf) file and the single-nucleotide polymorphism (SNP) file.

### Data analysis

#### Genetic diversity and genetic differentiation

The genetic diversity was estimated through the nucleotide diversity (Pi) measure proposed by Nei (1987) and implemented by the software DnaSP v6 [54]. The estimation was based on the sequence variation information stored in the variant call format (vcf) retrieved from the ipyrad alignment.

The underlying degree of genetic structure was determined through the Bayesian clustering approach implemented by the software STRUCTURE v2.3.4 [55]. The substructure was estimated from the set of 65 nuclear loci. Posterior probabilities for the ancestry model and allele frequency model parameters were estimated under the admixture model and the correlated allele frequencies model respectively. The MCMC process estimation was conducted using a burn-in of 1,000,000 followed by 100,000 chains for each of the 10 replicas assigned to a range of 1 to 9 K assumed groups. The most probable K value was estimated using the Evanno method [56] implemented in STRUCTURE HARVESTER [57] and the corresponding individual membership coefficients matrix was used as input file in STRUCTURE PLOT [58] to visualize the degree of genetic differentiation among groups.

Genetic structure was further evaluated through a global analysis of molecular variance (AMOVA) using the software Arlequin v3.5 [59]. The analysis was performed based on a weighted average over all loci and confidence intervals for the fixation index were calculated by bootstrapping with 20,000 replicates.

#### Phylogenetic network and divergence times

To visualize the phylogenetic relationships among and within populations, a phylogenetic network was constructed using the Neighbor-Net algorithm implemented by the software SplitsTree5 [60]. The relationships were estimated using the linked SNPs file. The Neighbor-Net method consists of the agglomeration of weighted collection of splits or partition of the set of taxa which constitute the building blocks of a phylogenetic tree and provides the visualization of the space of feasible trees. Thereof, constitutes a useful tool for the representation of the relationships of taxa in which the underlying evolutionary history may not be treelike due to processes such as recombination, hybridization, gene conversion and gene transfer [61].

To relate the genetic divergence among populations to pre-Pleistocene and Pleistocene events, calibrated time trees were calculated under the multispecies coalescent model implemented in StarBEAST2 [62]. StarBEAST2 models the incomplete lineage sorting process between and within species with no recent history of gene flow [63] and allows variation in substitution rates of different genes and species by using gene tree relaxed clock models [62]. The time trees were calculated from a selection of 36 polymorphic nuclear loci (Additional file 3) of approximately 140 bp. The best DNA substitution model (Table 1 in Additional file 4) and gamma rate heterogeneity for each locus were determined using jModelTest v2.1.10 [64] through the implementation of the Akaike information criterion (AIC). The corresponding site models were set with four rate categories and an uncorrelated lognormal molecular clock was established for the clock model. The constant populations model was selected for the population model parameter and the Calibrated Yule model for the tree prior. The time trees were calibrated using a secondary calibration point due to the absence of paleontological information to constrain a minimum age of divergence for *C. pentadactylon.* This calibration point derives from a dated phylogeny for the Malvaceae family, which was used to infer divergence dates and diversification rates within the *Theobroma* genus [40]. The resulting estimated age of divergence between *C. pentadactylon* and its sister genus *Fremontodendron* was on the order of 5 Ma. This was set as the median age for the informative lognormal prior with a standard deviation of 0.01. The MCMC chain length was 180,000,000 and logged every 5000. The log files were analyzed with Tracer v1.7.1 for parameter convergence. The burn-in was set to remove 10 % of the tree files before the generation of a maximum clade credibility tree with median heights in TreeAnnotator.

#### Demographic and evolutionary history

To assess changes in population size over time and determine their correspondence to pre-Pleistocene or Pleistocene events, a Coalescent Bayesian Skyline Plot (BSP) was estimated for each group defined in STRUCTURE and later defined as separate populations, using BEAST v.2.5.2. Linked SNPs data were set as input files. The best substitution model for each set of SNPs was determined using jModelTest through the implementation of the AIC (Table 2 in Additional file 4). The corresponding site models were set with four rate categories and a strict molecular clock was established for the clock model. The time scale was calibrated with the divergence age of 0.873 Ma previously estimated and a standard deviation of 0.2 under a lognormal distribution. The MCMC chain length was set to 10,000,000 and to 15,000,000 for the western and eastern population respectively and logged every 1000. The log files were analyzed in Tracer for parameter convergence.

The population history of *C. pentadactylon* was inferred from an approximate Bayesian computation (ABC) in which evolutionary scenarios were simulated and compared through posterior probabilities using the DIYABC v2.1.0 [65] software. The linked SNPs data file was used to simulate 1 million datasets per scenario. The evolutionary scenarios were built considering the genetic structure and StarBEAST2 analyses: (i) two populations (Pop1 and Pop2) have diverged simultaneously from an ancestral population at t1 (scenario1), (ii) Pop2 (Eastern population) diverged from Pop1 (Western population) at t1 (scenario 2), (iii) Pop1 diverged from Pop2 at t1 (scenario 3), (iv) Pop2 derived from few western immigrants at t2 and diverged at t1 (scenario 4, and (vi) Pop1 derived from few eastern immigrants at t2 and diverged at t1 (scenario 5).

Posterior probabilities of scenarios were assessed using a polychotomic weighted logistic regression on 1% of the simulated datasets. The fit between the simulated and observed datasets was evaluated through the implementation of a model checking procedure based on a principal component analysis (PCA). Confidence in scenario choice was assessed by means of the simulation of 500 pseudo-observed datasets (PODs) under each scenario to estimate Type I and Type II error rates.

#### Distribution modelling

The potential distribution of *C. pentadactylon* for the present day, Mid-Holocene (Mid-HLC, 6 Kyr), Last Glacial Maximum (LGM, 22 Kyr) and Last Interglacial (LIG, 130 Kyr) was predicted using Maxent 3.4.1 [66]. The georeferenced sampling locations were used as occurrence data in addition to data retrieved (15 March 2017) from the Global Biodiversity Information Facility (GBIF; *Chiranthodendron pentadactylon* Larreat in GBIF Secretariat (2019). GBIF Backbone Taxonomy. Checklist dataset https://doi.org/10.15468/39omei), TROPICOS (http://legacy.tropicos.org/Name/3900594, Missouri Botanical Garden, St. Louis, MO, USA; available from: http://www.tropicos.org) and REMIB (http://www.conabio.gob.mx/remib/doctos/remib_esp.html) databases and represented 152 unique localities after data cleansing including the removal of duplicate points. The models were built from 9 climate layers selected through the jackknife method. These had a 2.5 arc minute spatial resolution and were acquired from the WorldClim database [67]. Layers for the LGM and the Mid-HLC were obtained from two paleoclimate models (CCSM and MIROC) that simulate differing climatic predictions [68]. Distribution models were built with 10 replicates using the default settings and 25% of the occurrence data was selected for model validation.

## Supplementary information

**Additional file 1.** Taxon sampling, geographic location and voucher information. The two specimens collected as vouchers are deposited in the herbarium of the Universidad Autónoma de Aguascalientes (HUAA).

**Additional file 2.** RADSeq de novo assembly stats of raw sequences.

**Additional file 3.** Sequence matrix of 36 polymorphic nuclear loci.

**Additional file 4.** Nuclear loci and SNPs data sets substitution models.

**Additional file 5.** Evanno method results from STRUCTURE HARVESTER.

## Data Availability

The data that support the findings of this study is openly available in the Dryad Digital Repository: 10.5061/dryad.dfn2z34w9

## Abbreviations

ABC: Approximate Bayesian Computation
AIC: Akaike information criterion
AMOVA: Analysis of molecular variance
BSP: Bayesian skyline plot
CCSM: Community climate system model
GBIF: Global biodiversity information facility
LGM: Last glacial maximum
LIG: Last interglacial
MCMC: Markov Chain Monte Carlo
Mid-HLC: Mid-Holocene
MIROC: Model for interdisciplinary research on climate
MRCA: Most recent common ancestor
NCBI: National Center for Biotechnology Information
RADSeq: Restriction-site associated DNA sequencing
ROC: Receiver operating characteristic curve
SDM: Species distribution modeling
