# Correction to: Nuclear phylogeography of the temperate tree species *Chiranthodendron pentadactylon* (Malvaceae): Quaternary relicts in Mesoamerican cloud forests

**DOI:** 10.1186/s12862-020-01655-y

**Published:** 2020-07-31

**Authors:** Diana Gabriela Hernández-Langford, María Elena Siqueiros-Delgado, Eduardo Ruíz-Sánchez

**Affiliations:** 1 Departamento de Biología, Herbario UAA, Centro de Ciencias Básicas, Edificio 132, Universidad Autónoma de Aguascalientes, Av, Universidad No. 940, Ciudad Universitaria, 20131 Aguascalientes, Aguascalientes México; 2 Departamento de Botánica y Zoología, Centro Universitario de Ciencias, Biológicas y Agropecuarias, Universidad de Guadalajara, Camino Ing. Ramón, Padilla Sánchez 2100, Nextipac, 45200 Nextipac, Zapopan, Jalisco México

**Correction to: BMC Evolutionary Biology (2020) 20:44**

**https://doi.org/10.1186/s12862-020-01605-8**

Following publication of the original article [1], the authors notified us that the legend for Fig. 1 is incorrect (the order of the names is misplaced). The correct legend is presented below:

Fig. 1 STRUCTURE bar plot showing the individual membership coefficients assigned to each K number of groups (K = 2) defined as populations. The individuals are classified according to their sampling site: 1) Carrizal de Bravo, Guerrero. (2) San Mateo Río Hondo, Oaxaca. (3) Motozintla, Chiapas. (4) Tacaná volcano, Chiapas. (5) San Cristóbal de las Casas, Chiapas. (6) Acatenango volcano, Chimaltenango. (7) Chimaltenango Quetzaltenango. (8) Totonicapán. (9) Sierra de los Cuchumatanes, Huehuetenango.
