# Supplementary material for: Nuclear phylogeography of the temperate tree species Chiranthodendron pentadactylon (Malvaceae): Quaternary relicts in Mesoamerican cloud forests
Source: BMC Evol Biol. 2020 Apr 19;20:44. doi: 10.1186/s12862-020-01605-8 (PMC7168997; doi:10.1186/s12862-020-01605-8)
Supplement: Supplementary file 1 — Additional file 1. Taxon sampling, geographic location and voucher information. The two specimens collected as vouchers are deposited in the herbarium of the Universidad Autónoma de Aguascalientes (HUAA). [file 12862_2020_1605_MOESM1_ESM.docx]

**Taxon sampling, geographic location and voucher information**

| Location | Code | *n* | Altitude (masl) | Latitude (N) | Longitude (W) | Voucher information |
| --- | --- | --- | --- | --- | --- | --- |
| México, Guerrero, Leonardo Bravo | Gue | 10 | 2607 | 17º 36' 8.64" | 99º 50' 9.6" |  |
| México, Oaxaca, San Mateo Río Hondo | Oax | 10 | 2601 | 16º 10' 6.96" | 96º 30' 6.84" | Glez-Adame, Glez Gallegos 2829 (HUAA) |
| México, Chiapas, Motozintla | Chi1 | 5 | 2258 | 15º 22' 53.18" | 92º 19' 43.72" |  |
| México, Chiapas, Unión Juárez, Tacaná volcano | Chi2 | 5 | 2222 | 15º 5' 57.88" | 92º 6' 12.35" |  |
| México, Chiapas, San Cristóbal de las Casas | Chi3 | 5 | 2342 | 16º 40' 10" | 92º 33' 47" |  |
| Guatemala, Chimaltenango, Acatenango volcano | GTM1 | 10 | 2468 | 14º 32' 23.21" | 90º 52' 27.59" | Hdz-Langford, MV n/n (HUAA) |
| Guatemala, Chimaltenango-Quetzaltenango | GTM2 | 9 | 2284 | 14º 44' 7.26" | 90º 57' 54.48" |  |
| Guatemala, Totonicapán, Pologua | GTM3 | 5 | 2790 | 15º 2' 43.98" | 91º 29' 58.13" |  |
| Guatemala, Huehuetenango, Sierra de los Cuchumatanes | GTM4 | 10 | 2904 | 15º 34' 20.21" | 91º 27' 16.13" |  |
