# Supplementary material for: Nuclear phylogeography of the temperate tree species Chiranthodendron pentadactylon (Malvaceae): Quaternary relicts in Mesoamerican cloud forests
Source: BMC Evol Biol. 2020 Apr 19;20:44. doi: 10.1186/s12862-020-01605-8 (PMC7168997; doi:10.1186/s12862-020-01605-8)
Supplement: Supplementary file 4 — Additional file 4. Nuclear loci and SNPs data sets substitution models. [file 12862_2020_1605_MOESM4_ESM.docx]

***Chiranthodendron pentadactylon***

**Best substitution models**

| **Analysis:** Estimation of divergence times | | |
| --- | --- | --- |
|  | | |
| **Nuclear locus** | **jModeltest2** | **StarBEAST2** |
| **1** | TPM1 | HKY+G |
| **2** | F81 | GTR |
| **3** | TrNef | TN93 |
| **4** | HKY + G | HKY |
| **5** | F81 | GTR* |
| **6** | K80 | HKY** |
| **7** | F81 | GTR* |
| **8** | JC | JC69 |
| **9** | TrNef | TN93** |
| **10** | TrN | TN93 |
| **11** | TPM3uf | HKY+I+G |
| **12** | TrNef | TN93** |
| **13** | F81 | GTR* |
| **14** | TPM2uf | HKY |
| **15** | TrN | TN93 |
| **16** | HKY | HKY |
| **17** | F81 | GTR* |
| **18** | TrN+I | TN93 |
| **19** | GTR | GTR |
| **20** | TPM2uf | HKY |
| **21** | TIM3 | GTR* |
| **22** | TPM3uf | HKY+I+G |
| **23** | HKY | HKY |
| **24** | TPM1uf + G | HKY |
| **25** | TPM2uf | HKY |
| **26** | TIM1+I+G | GTR* |
| **27** | HKY+I | HKY |
| **28** | TIM2+I | GTR* |
| **29** | JC | JC69 |
| **30** | TPM2uf+G | HKY |
| **31** | TPM1uf | HKY |
| **32** | F81 | GTR* |
| **33** | TPM2uf | HKY |
| **34** | F81 | GTR* |
| **35** | K80 | HKY** |
| **36** | HKY | HKY |

**Table 1.** Best substitution model estimated for each nuclear locus using jModeltest2 and their equivalent in StarBEAST2.

*Operators turned off. **Base frequencies set to equal.

| Analysis:  Bayesian skyline plots | | |
| --- | --- | --- |
| SNP set | **jModeltest2** | **BEAST2** |
| Western population | GTR+G | GTR |
| Eastern population | SYM+G | GTR** |

**Table 2.** Best substitution model estimated for each set of SNPs using jModeltest2 and their equivalent in BEAST2.

**Base frequencies set to equal.
