# Supplementary material for: Nuclear phylogeography of the temperate tree species Chiranthodendron pentadactylon (Malvaceae): Quaternary relicts in Mesoamerican cloud forests
Source: BMC Evol Biol. 2020 Apr 19;20:44. doi: 10.1186/s12862-020-01605-8 (PMC7168997; doi:10.1186/s12862-020-01605-8)
Supplement: Supplementary file 5 — Additional file 5. Evanno method results from STRUCTURE HARVESTER. [file 12862_2020_1605_MOESM5_ESM.docx]

**Evanno method results from STRUCTURE HARVESTER**

*The number of K groups (K = 2) with the highest likelihood is determined by the maximum value of Delta K. This value is related to the second-degree derivative of the probability logarithm of the data with respect to the number of K groups.

| K | Reps | Mean LnP(K) | Stdev LnP(K) | Ln'(K) | \|Ln''(K)\| | Delta K |
| --- | --- | --- | --- | --- | --- | --- |
| 1 | 10 | -3025.96 | 0.0516 | - | - | - |
| 2 | **10** | **-1824.86** | **1.1974** | **1201.1** | **1120.62** | **935.874139** |
| 3 | 10 | -1744.38 | 7.3676 | 80.48 | 26.07 | 3.538458 |
| 4 | 10 | -1689.97 | 6.7277 | 54.41 | 90.03 | 13.381931 |
| 5 | 10 | -1725.59 | 234.235 | -35.62 | 69.09 | 0.29496 |
| 6 | 10 | -1692.12 | 84.0963 | 33.47 | 24.77 | 0.294543 |
| 7 | 10 | -1683.42 | 27.2742 | 8.7 | 71.94 | 2.637662 |
| 8 | 10 | -1746.66 | 275.1114 | -63.24 | 116.71 | 0.424228 |
| 9 | 10 | -1693.19 | 57.6657 | 53.47 | - | - |
